# Evaluation of Antioxidant Properties in Cereals: Study of Some Traditional Italian Wheats

**DOI:** 10.3390/foods4030391

**Published:** 2015-09-07

**Authors:** Alessandra Durazzo, Gaetana Casale, Valentina Melini, Giuseppe Maiani, Rita Acquistucci

**Affiliations:** Consiglio per la ricerca in agricoltura e l’analisi dell’economia agraria—Centro di ricerca per gli alimenti e la nutrizione, Via Ardeatina 546, 00178 Roma, Italy; E-Mails: gaetana.casale@entecra.it (G.C.); valentina.melini@entecra.it (V.M.); giuseppe.maiani@entecra.it (G.M.); rita.acquistucci@entecra.it (R.A.)

**Keywords:** antioxidant properties, total polyphenol content, cereals, traditional Italian wheat

## Abstract

Whole grain cereals contain a wide range of phytochemicals and it is often difficult to ascribe protective effects on metabolic health to any one particular constituent. The interactions among bioactive components, which contribute highly to the total antioxidant capacity of cereals, represent the first step in the evaluation of food potential health benefits. This research focused on the determination of antioxidant properties in grains and whole flours of some traditional Italian wheats. Results showed that hydrolysable polyphenols in grains are 85% of total polyphenols and contribute 95% of the total antioxidant properties, which indicates that hydrolysable polyphenols represent an important fraction of polyphenols in cereals. The distinction between extractable and non-extractable antioxidants was shown to be of paramount importance for an adequate determination of antioxidant capacity in cereals and represents a key element in the definition of potential nutritional value of the food matrix under consideration.

## 1. Introduction

In the last decades, several investigations have underlined the inverse association between whole grain consumption and risk of chronic diseases, such as cardiovascular diseases and cancer [1,2,3,4,5]. Wholegrain cereals contain, in fact, a high amount of bioactive components represented by insoluble and soluble fiber, vitamins, minerals, unsaturated fatty acids, tocotrienols, tocopherols, lignans, phenols, *etc.* [6]. Evaluation of the relative content of bioactive compounds and assessment of interactions between these molecules and other food matrix nutrients represent the main step in total antioxidant capacity determination, and consequently in evaluation of potential health benefits. However, the structural diversity of each compound, in addition to possible interactions thereof, different mechanisms of action as well as biological role, makes difficult to assess a reliable procedure to evaluate antioxidant activity.

The three fundamental steps in evaluation of antioxidant properties are the extraction procedure, the antioxidant capacity measurement and the expression of results [7]. Extraction is quite a key issue, as it is typically undertaken through different procedures and so far some authors [8,9] reported the use of different solvents, *i.e.*, ethanol, methanol and acetone and/or mixtures thereof. It emerged, nevertheless, that an acid mixture of methanol/water affords the highest level of antioxidant compounds extraction capacity [10]. However, recent studies have also performed alkaline hydrolysis, acid hydrolysis or enzymatic digestion [11].

Antioxidants exist as easily extractable compounds (free forms), solubilized by aqueous-organic solvent mixtures, and as less extractable compounds (bound forms), which remain in the residue after the aqueous-organic extraction [12]. In cereals, non-extractable antioxidants represent an important fraction, as demonstrated by several investigations that found in grains more antioxidant compounds than previously thought, due to the relatively high amount of bound compounds [13,14].

As regards the bound fraction, it has recently got attention by researchers because most antioxidant bioactive compounds in grains can survive passage through the gastrointestinal tract and reach the colon intact, where they thus produce substantial antioxidant activity [15,16]. In the colon, fiber is fermented and some bioactive compounds, which have antioxidant activity, are released.

In previous studies a specific procedure to evaluate extractable and bound phenolic compounds was assessed [17,18].

This research focused on the antioxidant properties of Italian wheats grown in small scale in a mountainous region; in particular, Ruscia is an example of Italian traditional durum wheat, native of Sicily (Southern Italy), and now cultivated in Abruzzo Region due to its adaptability to mountainous areas, within the National Park of Gran Sasso.

To fully characterize samples, some technological and chemical parameters were also considered besides the characterization of antioxidant properties.

## 2. Experimental Section

### 2.1. Sampling

Grains were supplied by a farm located at Castelvecchio Subequo (Italy), in the province of L’Aquila, at an altitude of 465–642 m above sea level. According to the genetic origin, they were labelled and are here reported as “Ruscia 1” and “Ruscia 2”. Ruscia 1 is a mixture of one durum wheat (*Ruscia*) and two cultivars of soft wheat, which grow wild in this area. “Ruscia 2” is a durum wheat cultivar (*Ruscia*) originating from Ruscia 1 after four years of phenotypic selection.

Grains were milled by a water-cooled mill (Janke and Kunkel IKA LabortechniK, Stanfen, Germany) and then used for the determination of both chemical composition and antioxidant properties.

Whole grains were tempered for 24 h to 15.5% moisture and whole flours were obtained by milling each sample in an experimental mill (MLU-202 Buhler, Switzerland) equipped with three breaks and three reduction rolls and six screens. Antioxidant properties were evaluated on whole flours as well.

### 2.2. Chemicals

Common reagents and standards were purchased from Sigma-Aldrich Srl (Milan, Italy), Extrasynthèse (Genay, France), Carlo Erba (Milan, Italy) and BDH Laboratory Supplies (Poole, UK). Double-distilled water (Millipore, Milan, Italy) was used throughout the study.

### 2.3. General Chemical Analysis

Grain impurities (broken grains, heat-damaged grains, shriveled grains, vegetable impurities, mottled grains, spottled grains) were determined on 100 g seeds by visual evaluation and then removed. The grain fraction free from impurities was thus used to assess grain quality. Test weight (TW) was determined by a Shopper chondrometer equipped with 250 mL cylinder. Thousand Kernel Weight (KW) was determined by counting and weighing 1000 kernels. Moisture was reported according to the AACC standard method 55-31 [19].

Total proteins were determined by the Kjeldahl method according to the ICC standard method No. 105/2 [20] using 5.70 as specific conversion factor. Ash content was determined on the inorganic residue remaining after the incineration of the sample in a muffle furnace (Z-1200, Sinergica soluzioni S.r.l., Milan, Italy) at 900 °C according to ICC Standard No. 104/1 [20]. Moisture is reported as g/100 g fresh weight (fw), while protein and ash content are reported as g/100 g dry matter (dm).

### 2.4. Total Polyphenol Content (TPC) and Antioxidant Properties

Total polyphenols were extracted as described by Durazzo *et al.* [17] with slight modifications. Aqueous-organic extracts (extractable polyphenols) and their residues (non-extractable polyphenols) were isolated and studied. In residues, in particular, hydrolysable polyphenols, part of non-extractable polyphenols, were isolated and determined by following specific and suitable acid hydrolysis as reported below.

*Aqueous-organic extract.* About 3.0–3.5 g sample were placed in a test tube and 20 mL methanol/water (50:50 *v*/*v*), adjusted to pH = 2 by HCl, were added. Tubes were swirled at room temperature for 3 min, then mildly shaken for 1 h in a water bath at room temperature. Tubes were centrifuged at 2500× g for 10 min and supernatants were recovered. Twenty milliliters acetone/water (70:30 *v*/*v*) were added to residue, then swirling, shaking and centrifugation were repeated. Methanolic and acetonic extracts were combined and centrifuged at 2800× g for 15 min. The resulting supernatant was transferred into tubes and directly used for the determination of antioxidant properties and total polyphenol content.

*Residue*. The residue that remained after the solvent extraction was left in a ventilated and heated apparatus (max temperature 25 °C) until dried. About 200–300 mg residue were mixed with 20 mL methanol and 2 mL concentrated sulfuric acid (18 M). Samples were gently stirred for 1 min and left under shaking at 85 °C for 20 h in a water bath.

Samples were centrifuged (2500× g for 10 min) and supernatant recovered. After two washings with minimum volume of distilled water, suspension was centrifuged once more, when necessary, and the final volume was taken up to 50 mL. Tubes were centrifuged at 2800× g for 20 min and the resulting supernatant was used for the determination of antioxidant properties and total polyphenol content.

*Ferric Reducing Antioxidant Power (FRAP).* This determination was carried out according to the methods by Benzie and Strain [21] and Pulido *et al.* [22] by means of a Tecan Sunrise^®^ plate reader spectrophotometer. The method is based on the reduction of Fe^3+^-TPTZ (2,4,6-tripyridyl-s-triazine) complex to ferrous at low pH.

*Total Polyphenol Content (TPC).* This assay was carried out by using the Folin-Ciocalteau procedure [23]*.* Briefly, appropriately diluted extracts were oxidized with Folin-Ciocalteau reagent, and the reaction was neutralized with sodium carbonate. The absorbance of the resulting blue color was measured at 760 nm against an appropriate blank after 2 h of reaction at room temperature. Gallic acid was used as standard.

### 2.5. Statistical Analysis

Data are here shown as mean values of replicate measurements (*n* = 3) with standard deviation. Statistica for Windows (Statistical package; release 4.5; StatSoft Inc., Vigonza, PD, Italy) was used to perform Student’s *t*-test. *p*-Values < 0.05 were considered significant.

## 3. Results and Discussion

### 3.1. General Chemical Analysis

**Figure 1:**
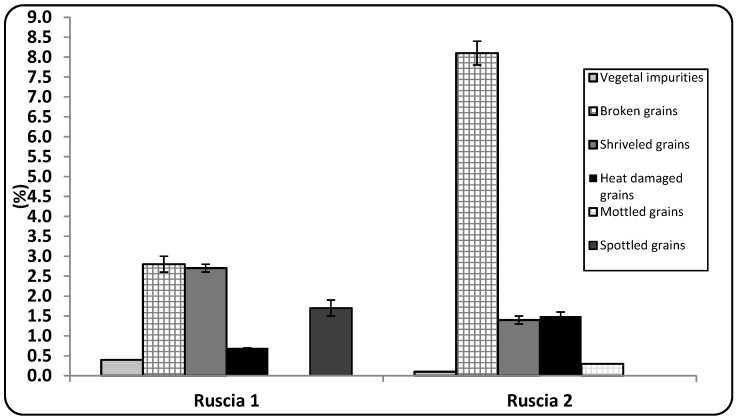
Kernel impurities (%).

The inspection of grains to check impurities (*i.e.*, broken/cracked grains, shriveled grains, rusty or heated grains, mottled grains, spottled grains) is a necessary control procedure to verify wheat quality, before proceeding to the milling phase. The analyzed samples resulted well developed and not sprouted. As reported in Figure 1, on a 100 g grain basis, 92% Ruscia 1 grains and 89% Ruscia 2 grains were sound, that is, grains showed to be clean, well mature, free of kernel damage and foreign material.

Some physical parameters of grains (test weight, 1000 kernel weight), as well as some chemical characteristics (moisture, protein, ash) were reported in Table 1.

As concerns physical parameters, significant differences were found among grain samples. Our results are confirmed by Troccoli *et al.* [24] that reported in a paper on sixteen durum wheat cultivars grown in two locations of Southern Italy, variation of TW from 77.0 to 82.2 kg/hL in 1993 and from 79.0 to 84.1 kg/hL in 1994.

Regarding the main chemical parameters, significant differences were observed in protein and ash content. These results are in accordance with literature data [25,26], e.g., Acquistucci *et al.* [25] reported that protein content varied from 12.4 to 17.3 g/100 g dm and ash content from 1.79 to 2.15 g/100 g dm in durum wheat cultivars, whereas in soft wheat cultivars protein content ranged from 11.0 to 16.8 g/100 g dm and ash content from 1.56 to 2.19 g/100 g dm.

**Table 1:** Physical and chemical parameters of grains*.

Sample	TW (kg/hL)	1000 KW (g)	Moisture (g/100 g)	Protein (g/100 g dm)	Ash (g/100 g dm)
RUSCIA 1	74.6 ± 0.1^a^	41.4 ± 0.1^a^	12.4	14.1 ± 0.0^a^	1.77 ± 0.01^a^
RUSCIA 2	78.2 ± 0.1^b^	49.8 ± 0.1^b^	11.6	14.8 ± 0.0^b^	1.99 ± 0.00^b^

* Data are expressed as mean ± s.d. of replicate measurements (*n* = 3); Student’s *t*-test: by column, means followed by different letters are significantly different (*p* < 0.05).

### 3.2. Antioxidant Properties and Total Polyphenol Content

In Table 2, Total Polyphenol Content (TPC) and Ferric Reducing Antioxidant Power (FRAP) values were reported for grains and whole wheat flours. Values refer to aqueous-organic extracts and residues after aqueous-organic treatment.

In grains and flours, the lowest TPC and FRAP values, both in aqueous-organic extracts and residues, were obtained for the Ruscia 2 samples that show characteristics which are peculiar of durum wheat, as reported by some authors [27].

In this investigation, it emerged that hydrolysable polyphenols represent the predominant fraction. In grains they accounted for 85% of total polyphenols—calculated as percentage of TPC value in residue *vs.* sum of TPC values of aqueous-organic extract and residue—and contributed 95% of the total antioxidant properties—calculated as percentage of FRAP value in residue *vs.* sum of FRAP values of aqueous-organic extract and residue. In flours, hydrolysable polyphenols exhibited 89% for Ruscia 1 and 92% for Ruscia 2, and contributed 97% and 98%, respectively, of the total antioxidant properties.

**Table 2:** Total Polyphenol Content (TPC) and Ferric Reducing Antioxidant Power (FRAP) values in aqueous-organic extracts and their residues in grains and flours*.

Sample	TPC (mg/100 g dm)				FRAP (µmol/g dm)		
	Grain	Flour	*p* value^Ω^	Grain		Flour	*p* value^Ω^
	**Aqueous-Organic Extract**						
RUSCIA 1	175.85 ± 32.58^b^	145.60 ± 8.86^b^	n.s.	5.28 ± 0.15^b^		4.44 ± 0.26^b^	0.0001
RUSCIA 2	149.46 ± 23.73^a^	102.07 ± 5.55^a^	0.0001	4.46 ± 0.41^a^		2.18 ± 0.10^a^	0.0001
	**Residue**						
RUSCIA 1	1036.19 ± 57.56^b^	1161.39 ± 17.37	0.05	96.79 ± 8.29^b^		170.71 ± 1.54^b^	0.0001
RUSCIA 2	952.54 ± 16.14^a^	1142.19 ± 46.07	0.0001	85.45 ± 4.36^a^		133.09 ± 11.53^a^	0.0001

* Data are expressed as mean ± s.d. of replicate measurements (*n* = 3); Student’s *t*-test: by column, means followed by different letters are significantly different (*p* < 0.05). ^Ω^ Student’s *t*-test; n.s. = not significant.

The hydrolysable polyphenols, isolated in the residue after strong acid hydrolysis, belong to several classes of bioactive components (hydrolysable tannins, phenolic acids and hydroxycinnamic acids) and these compounds are linked to carbohydrates and proteins by covalent bonds, hydrogen bonds and/or hydrophobic interactions [15].

Our results match the main findings of several researches demonstrating that these compounds represent a significant fraction in cereals [14,18,28] as much as other food groups, *i.e.*, fruits, vegetables, *etc.*, and also exhibit appreciable antioxidant properties [29]. Durazzo *et al.* [18] themselves reported, when studying total polyphenols and antioxidant properties in Italian soft wheat grains, that TPC values varied from 165.57 to 183.75 mg/100 g dm in aqueous-organic extracts, and from 1084.42 to 1325.32 mg/100 g dm in residues, whereas the range for FRAP values was 6.34–6.47 µmol/g dm in aqueous-organic extracts and 85.06–109.12 µmol/g dm in residues.

Our findings also contribute to filling a gap in this field, as antioxidant properties have been generally underestimated and more studies are required. In addition, recent investigations studied the potential connection between non-extractable polyphenols and dietary fiber: these compounds significantly contribute to possibly specific health properties associated with dietary fiber [30,31].

As reported in Table 2, the milling process affects TPC and FRAP: in comparison with grains, a significant decrease was observed in aqueous-organic extracts for flours, while a significant increase was found in the residues. The increase of FRAP and TPC values in residue is likely correlated to a more efficient extraction of bound bioactive compounds due to the fact that solvent can penetrate more in depth following the major available surface of milled particles.

These results thus confirm how the milling procedure is a key factor in the evaluation of antioxidant properties of cereal matrices [32].

## 4. Conclusions

Results obtained after evaluation of antioxidant compounds properties in the Ruscia grain and flour samples underlined that the evaluation of the extractable and non-extractable compounds in cereals is essential for an appropriate determination of antioxidant capacity in cereals. Moreover, the presence of appreciable antioxidant properties in the ancient Italian wheat variety under consideration—Ruscia—potentially contributes to valorizing and promoting traditional wheat varieties, as well as supporting biodiversity and sustainable agriculture systems.
